# Supplementary material for: Maintenance of homeostatic plasticity at the Drosophila neuromuscular synapse requires continuous IP3-directed signaling
Source: eLife. 2019 Jun 10;8:e39643. doi: 10.7554/eLife.39643 (PMC6557630; doi:10.7554/eLife.39643)
Supplement: Supplementary file 1. — Genotypes and/or conditions are denoted. For GAL4 drivers, ‘Pre +Post Gal4’ denotes a genetic combination of elaV(C155)-Gal4/Y; Sca-Gal4/+; BG57-Gal4/+. Average values ± SEM are presented for each electrophysiological parameter, with n = number of NMJs recorded. Values include miniature excitatory postsynaptic potential (mEPSP) amplitude, mEPSP frequency (Freq), excitatory postsynaptic potential (EPSP) amplitude, quantal content (QC), and QC corrected for non-linear summation (NLS). *p<0.05, **p<0.01, ***p<0.001 vs. unchallenged control. [file elife-39643-supp1.docx]

**Supplementary File 1**

| **FIGURE 1** | | | | | | | | |
| --- | --- | --- | --- | --- | --- | --- | --- | --- |
| **Condition** | **Genotype or Reagent** | **mEPSP (mV)** | **mEPSP freq. (Hz)** | **EPSP (mV)** | **V_m_ (mV)** | **QC** | **NLSC QC** | **n** |
| Driver control | *Pre + Post-Gal4* | 0.83 ± 0.03 | 1.0 ± 0.1 | 40.4 ± 1.4 | -69.0 ± 1.2 | 48.6 ± 1.1 | 100.2 ± 3.7 | 13 |
| PhTox +  Driver control | *Pre + Post-Gal4* >>  + PhTox | 0.59 ± 0.04 | 0.5 ± .01 | 40.5 ± 1.0 | -67.0 ± 0.9 | 70.5 ± 4.3 *** | 150.6 ± 10.7 *** | 11 |
| *GluRIII* | *Pre + Post-Gal4* >> *UAS-GluRIII RNAi*/+ | 0.64 ± 0.03 | 0.4 ± 0.0 | 37.5 ± 1.2 | -70.1 ± 1.2 | 58.9 ± 2.1 ***  (vs. Driver control) | 110.6 ± 5.7 | 13 |
| *GluRIII* | *Pre + Post-Gal4* >> *UAS-GluRIII RNAi*/+  + PhTox | 0.37 ± 0.01 | 0.2 ± 0.0 | 29.6 ± 1.0 | -65.8 ± 0.8 | 80.1 ± 3.5 ***  (vs. *GluRIII* alone) | 133.2 ± 7.6 | 15 |
| wild type |  | 0.78 ± 0.02 | 2.0 ± 0.1 | 36.6 ± 0.7 | -67.1 ± 0.6 | 48.3 ± 1.3 | 93.3 ± 3.0 | 57 |
| wild type | + PhTox | 0.53 ± 0.02 | 0.8 ± 0.1 | 33.3 ± 0.7 | -64.4 ± 0.3 | 65.1 ± 2.8 *** | 119.7 ± 6.3 *** | 24 |
| *Plc21C*  (*GluRIII* cont) | *Pre + Post-Gal4* >> *GD11359* | 0.80 ± 0.04 | 1.3 ± 0.1 | 34.9 ± 1.4 | -67.4 ± 1.4 | 44.7 ± 2.8 | 83.7 ± 6.9 | 14 |
| *Plc21C* | *Pre + Post-Gal4* >> *GD11359/+*  *GluRIII RNAi/+* | 0.55 ± 0.01 | 0.3 ± 0.0 | 29.6 ± 1.0 | -66.0 ± 0.7 | 53.9 ± 0.5 *  (vs. *Plc21C* *RNAi*) | 89.4 ± 5.0 | 17 |
| *Plc21C* | *Pre + Post-Gal4* >> *GD11359/+*  *GluRIII RNAi/+*  + PhTox | 0.42 ± 0.01 | 0.2 ± 0.1 | 27.3 ± 1.7 | -64.9 ± 1.2 | 64.9 ± 4.1 *  (vs. *Plc21C + GluRIII* *RNAi*) | 105.1 ± 10.4 | 11 |
| *Plc21C*  (PhTox cont) | *Pre + Post-Gal4* >> *GD11359* | 0.80 ± 0.04 | 0.7 ± 0.1 | 31.6 ± 0.9 | -66.0 ± 0.9 | 40.6 ± 2.0 *** | 69.8 ± 6.5 | 14 |
| *Plc21C* | *Pre + Post-Gal4* >>  *GD11359/+*  + PhTox | 0.51 ± 0.01 | 0.3 ± 0.1 | 30.9 ± 1.7 | -66.5 ± 0.9 | 61.1 ± 3.7 *** | 106.8 ± 9.5 ** | 14 |
