# Supplementary material for: Maintenance of homeostatic plasticity at the Drosophila neuromuscular synapse requires continuous IP3-directed signaling
Source: eLife. 2019 Jun 10;8:e39643. doi: 10.7554/eLife.39643 (PMC6557630; doi:10.7554/eLife.39643)
Supplement: Supplementary file 2. — Genotypes and/or conditions are denoted. The data are split into two tables. The first table summarizes the screen data from Figure 2A. The second table summarizes the follow-up data examining the UAS-IP3-sponge reagent, including the homoeostatic block identified in the screen. Average values ± SEM are presented for each electrophysiological parameter, with n = number of NMJs recorded. Values include miniature excitatory postsynaptic potential (mEPSP) amplitude, mEPSP frequency (Freq), excitatory postsynaptic potential (EPSP) amplitude, quantal content (QC), and QC corrected for non-linear summation (NLS). *p<0.05, **p<0.01, ***p<0.001 vs. unchallenged control. [file elife-39643-supp2.docx]

**Supplementary File 2**

| **FIGURE 2 – SCREEN** | | | | | | | | |
| --- | --- | --- | --- | --- | --- | --- | --- | --- |
| **Condition** | **Genotype or Reagent** | **mEPSP (mV)** | **mEPSP freq. (Hz)** | **EPSP (mV)** | **V_m_ (mV)** | **QC** | **NLSC QC** | **n** |
| wild type | *w^1118^* | 0.88 ± 0.02 | 3.3 ± 0.2 | 38.3 ± 0.7 | -68.2 ± 0.7 | 44.1 ± 1.0 | 87.6 ± 2.7 | 52 |
| *GluRIIA* | *GluRIIA^SP16^* | 0.46 ± 0.02 | 0.9 ± 0.1 | 29.2 ± 1.0 | -68.7 ± 0.8 | 66.0 ± 3.1 *** | 107.3 ± 6.2 *** | 24 |
| *GluRIII RNAi* | *Pre + Post-Gal4* | 0.81 ± 0.02 | 2.0 ± 0.7 | 37.5 ± 1.3 | -68.3 ± 1.0 | 46.7 ± 1.7 | 91.9 ± 5.0 | 22 |
|  | *Pre + Post-Gal4 >>*  *GluRIII* RNAi/+ | 0.57 ± 0.02 | 0.3 ± 0.0 | 34.1 ± 0.8 | -67.6 ± 1.0 | 60.5 ± 1.9 *** | 108.3 ± 3.9 * | 17 |
| *CG7611* | *CG7611^EY07631^* | 0.93 ± 0.03 | 3.3 ± 0.3 | 28.3 ± 1.3 | -67.3 ± 0.9 | 30.9 ± 1.7 | 49.6 ± 3.4 | 12 |
|  | *GluRIIA^SP16^; CG7611^EY07631^* | 0.45 ± 0.02 | 1.6 ± 0.2 | 23.6 ± 1.7 | -67.1 ± 1.0 | 53.1 ± 4.4 *** | 79.6 ± 8.8 ** | 13 |
| *Mipp2* | *Pre + Post-Gal4 >>*  *TRiP.HMC03229* | 0.98 ± 0.04 | 1.1 ± 0.2 | 36.4 ± 1.5 | -69.8 ± 1.6 | 37.4 ± 1.5 | 69.5 ± 3.9 | 10 |
|  | *Pre + Post-Gal4 >>*  *TRiP.HMC03229 + GluRIII* RNAi | 0.65 ± 0.03 | 0.2 ± 0.0 | 33.7 ± 1.2 | -68.1 ± 1.2 | 52.5 ± 2.3 *** | 92.6 ± 4.7 ** | 9 |
| *Mipp2* | *Pre + Post-Gal4 >>*  *4317-R2* | 0.82 ± 0.03 | 0.8 ± 0.1 | 43.9 ± 1.7 | -71.4 ± 1.4 | 53.9 ± 2.3 | 118.9 ± 8.6 | 11 |
|  | *Pre + Post-Gal4 >>*  *4317-R2 + GluRIII* RNAi | 0.60 ± 0.03 | 0.4 ± 0.0 | 37.0 ± 1.6 | -71.7 ± 1.1 | 62.0 ± 2.0 * | 113.9 ± 5.1 | 10 |
| *Mipp2* | *Mipp2^KG01786^* | 0.97 ± 0.04 | 1.7 ± 0.2 | 39.0 ± 1.1 | -69.8 ± 1.1 | 40.8 ± 1.7 | 81.1 ± 4.5 | 15 |
|  | *Mipp2^KG01786^; GluRIIA^SP16^* | 0.52 ± 0.03 | 1.1 ± 0.2 | 29.6 ± 1.7 | -71.7 ± 1.1 | 57.2 ± 3.4 *** | 92.4 ± 6.9 | 16 |
| *slowpoke* | *slo^4^* | 0.83 ± 0.08 | 5.0 ± 0.1 | 36.3 ± 1.6 | -71.3 ± 1.0 | 47.7 ± 2.8 | 87.0 ±5.6 | 18 |
|  | *GluRIIA^SP16^; slo^4^* | 0.42 ± 0.02 | 1.7 ± 0.1 | 26.0 ± 1.7 | -70.3 ± 1.0 | 64.1 ± 5.2 * | 100.4 ± 9.4 | 22 |
| *slowpoke* | *slo^4^/slo^1^* | 0.93 ± 0.05 | 3.7 ± 0.4 | 35.9 ± 1.2 | -69.9 ± 0.9 | 40.1 ± 2.2 | 73.9 ± 4.5 | 19 |
|  | *GluRIIA^SP16^; slo^4^/slo^1^* | 0.38 ± 0.01 | 1.4 ± 0.2 | 25.4 ± 1.6 | -70.8 ± 0.9 | 67.5 ± 4.0 *** | 102.6 ± 8.7 *** | 18 |
| *Gβ13F* | *Gβ13F^KG08410^* | 0.97 ± 0.06 | 1.4 ± 0.2 | 26.9 ± 1.9 | -63.2 ± 0.4 | 28.1 ± 2.3 | 45.6 ± 4.9 | 8 |
|  | *GluRIIA^SP16^;* *Gβ13F^KG08410^* | 0.52 ± 0.01 | 2.6 ± 0.6 | 31.8 ± 1.5 | -66.2 ± 0.1 | 61.1 ± 2.6 *** | 106.0 ± 7.2 *** | 7 |
| *Gβ76C* | *Gβ76C^1^* | 0.99 ± 0.05 | 1.0 ± 0.1 | 39.6 ± 0.9 | -69.8 ± 1.1 | 40.6 ± 1.9 | 80.9 ± 4.0 | 7 |
|  | *GluRIIA^SP16^;* *Gβ76C^1^* | 0.50 ± 0.02 | 0.7 ± 0.1 | 29.6 ± 2.0 | -71.9 ± 2.6 | 59.4 ± 2.6 *** | 93.9 ± 6.5 | 8 |

| **Condition** | **Genotype or Reagent** | **mEPSP (mV)** | **mEPSP freq. (Hz)** | **EPSP (mV)** | **V_m_ (mV)** | **QC** | **NLSC QC** | **n** |
| --- | --- | --- | --- | --- | --- | --- | --- | --- |
| *Gβ76C* | *Pre + Post-Gal4 >>*  *GD 13785* | 0.73 ± 0.04 | 0.4 ± 0.0 | 41.1 ± 1.4 | -70.3 ± 0.9 | 57.9 ± 3.3 | 120.1 ± 7.8 | 13 |
|  | *Pre + Post-Gal4 >>*  *GD 13785*  *GluRIII* RNAi | 0.54 ± 0.02 | 0.3 ± 0.0 | 34.5 ± 1.4 | -69.3 ± 1.7 | 64.7 ± 0.7 | 115.6 ± 2.6 | 16 |
| *TkR86C* | *Pre + Post-Gal4 >>*  *TRiP.JF02160* | 1.00 ± 0.06 | 0.4 ± 0.1 | 36.7 ± 2.0 | -66.7 ± 0.8 | 38.5 ± 3.6 | 77.5 ± 9.8 | 10 |
|  | *Pre + Post-Gal4 >>*  *GluRIII* RNAi/ *TRiP.JF02160* | 0.78 ± 0.02 | 0.3 ± 0.0 | 41.2 ± 1.1 | -66.9 ± 1.5 | 52.6 ± 2.0 ** | 113.9 ± 5.7 ** | 8 |
| *mAChR-A* | *Pre + Post-Gal4 >>*  *TRiP.HMC02343* | 0.97 ± 0.03 | 1.1 ± 0.2 | 37.1 ± 1.1 | -66.3 ± 0.6 | 38.7 ± 1.5 | 76.2 ± 4.2 | 14 |
|  | *Pre + Post-Gal4 >>*  *TRiP.HMC02343 + GluRIII* RNAi | 0.71 ± 0.03 | 0.1 ± 0.0 | 34.1 ± 1.5 | -66.7 ± 0.8 | 48.5 ± 1.5 *** | 88.3 ± 4.3 * | 13 |
| *GABA-B-R1* | *Pre + Post-Gal4 >>*  *TRiP.HMC03388* | 1.00 ± 0.05 | 0.7 ± 0.1 | 39.6 ± 1.4 | -69.8 ± 0.7 | 40.1 ± 1.5 | 80.2 ± 3.9 | 7 |
|  | *Pre + Post-Gal4 >>*  *TRiP.HMC03388 + GluRIII* RNAi | 0.78 ± 0.02 | 0.3 ± 0.0 | 35.1 ± 0.8 | -66.5 ± 1.2 | 45.1 ± 1.4 * | 83.5 ± 3.1 | 8 |
| *PK2-R2* | *Pre + Post-Gal4 >>*  *TRiP.JF03209* | 1.02 ± 0.05 | 1.6 ± 0.5 | 36.0 ± 1.6 | -67.1 ± 1.0 | 35.7 ± 0.9 | 67.3 ± 2.4 | 8 |
|  | *Pre + Post-Gal4 >>*  *TRiP.JF03209 + GluRIII* RNAi | 0.80 ± 0.04 | 0.8 ± 0.1 | 35.2 ± 1.8 | -64.5 ± 0.8 | 44.2 ± 1.9 ** | 85.1 ± 5.6 * | 9 |
| *methuselah* | *Pre + Post-Gal4 >>*  *TRiP.GL01060* | 0.81 ± 0.03 | 1.1 ± 0.2 | 37.5 ± 1.5 | -68.2 ± 1.1 | 46.9 ± 2.0 | 91.7 ± 5.5 | 15 |
|  | *Pre + Post-Gal4 >>*  *TRiP.GL01060 + GluRIII* RNAi | 0.66 ± 0.03 | 0.4 ± 0.1 | 36.7 ± 1.3 | -70.5 ± 1.1 | 56.4 ± 1.9 ** | 105.3 ± 5.2 | 13 |
| *AdoR* | *Pre + Post-Gal4 >>*  *TRiP.JF02687* | 0.89 ± 0.05 | 1.5 ± 0.2 | 33.1 ± 1.7 | -69.3 ± 1.3 | 37.8 ± 2.5 | 65.8 ± 5.7 | 8 |
|  | *Pre + Post-Gal4 >>*  *TRiP.JF02687 + GluRIII* RNAi | 0.69 ± 0.03 | 0.6 ± 0.1 | 32.5 ± 1.9 | -67.6 ± 1.8 | 46.9 ± 2.4 * | 81.7 ± 5.9 | 9 |
| *PKC* | *Pre + Post-Gal4 >>*  *UAS-PKCi.B* | 0.79 ± 0.04 | 1.0 ± 0.2 | 35.7 ± 1.7 | -70.3 ± 1.5 | 45.9 ± 2.1 | 83.6 ± 5.2 | 14 |
|  | *Pre + Post-Gal4 >>*  *UAS-PKCi.B + GluRIII* RNAi | 0.62 ± 0.03 | 0.2 ± 0.0 | 33.5 ± 1.0 | -66.7 ± 1.2 | 55.4 ± 3.1 * | 100.8 ± 8.0 | 14 |
| *unc-13* | *Pre + Post-Gal4 >>*  *KK109346* | 1.12 ± 0.04 | 2.6 ± 0.2 | 41.6 ± 1.7 | -73.0 ± 1.4 | 37.5 ± 1.6 | 77.0 ± 4.7 | 15 |
|  | *Pre + Post-Gal4 >>*  *KK109346 + GluRIII* RNAi | 0.88 ± 0.03 | 0.8 ± 0.1 | 39.8 ± 1.2 | -68.6 ± 0.8 | 45.4 ± 1.1 *** | 92.7 ± 3.6 * | 15 |

| **Condition** | **Genotype or Reagent** | **mEPSP (mV)** | **mEPSP freq. (Hz)** | **EPSP (mV)** | **V_m_ (mV)** | **QC** | **NLSC QC** | **n** |
| --- | --- | --- | --- | --- | --- | --- | --- | --- |
| *iav* | *Pre + Post-Gal4 >>*  *TRiP.JF01904* | 0.89 ± 0.03 | 0.9 ± 0.1 | 35.9 ± 1.0 | -70.7 ± 1.2 | 40.6 ± 1.4 | 73.8 ± 3.3 | 14 |
|  | *Pre + Post-Gal4 >>*  *TRiP.JF01904 + GluRIII* RNAi | 0.70 ± 0.16 | 0.4 ± 0.1 | 32.1 ± 1.2 | -67.7 ± 1.0 | 45.6 ± 1.6 * | 78.7 ± 4.1 | 14 |
| *Slip1* | *Pre + Post-Gal4 >>*  *TRiP.HMC03268* | 0.70 ± 0.03 | 0.9 ± 0.1 | 31.2 ± 1.6 | -65.7 ± 0.6 | 45.2 ± 3.2 | 79.2 ± 8.0 | 11 |
|  | *Pre + Post-Gal4 >>*  *TRiP.HMC03268 + GluRIII* RNAi | 0.56 ± 0.03 | 0.3 ± 0.0 | 33.1 ± 1.9 | -67.7 ± 1.0 | 59.6 ± 3.1 ** | 105.7 ± 8.3 * | 9 |
| *mGluR* | *mGluR^MI02169^* | 1.01 ± 0.05 | 7.0 ± 0.8 | 36.0 ± 1.1 | -64.3 ± 0.7 | 36.0 ± 2.3 | 70.5 ± 5.9 | 6 |
|  | *GluRIIA^SP16^*;;*mGluR^MI02169^* | 0.45 ± 0.01 | 1.7 ± 0.9 | 28.7 ± 1.6 | -63.0 ± 0.7 | 64.4 ± 4.3 *** | 108.8 ± 11.7 | 7 |
| *Pka-R2* | *Pre + Post-Gal4 >>*  *TRiP.JF02759* | 0.79 ± 0.04 | 1.0 ± 0.1 | 35.0 ± 1.4 | -64.9 ± 0.5 | 44.7 ± 1.7 | 84.5 ± 4.4 | 8 |
|  | *Pre + Post-Gal4 >>*  *TRiP.JF02759* | 0.58 ± 0.03 | 2.7 ± 2.4 | 31.4 ± 1.9 | -64.8 ± 1.5 | 54.3 ± 2.6 ** | 94.6 ± 6.9 | 7 |
| *Gβ5* | *Pre + Post-Gal4 >>*  *TRiP.JF02941* | 1.15 ± 0.07 | 1.6 ± 0.2 | 39.4 ± 1.7 | -70.6 ± 2.2 | 35.0 ± 1.8 | 69.0 ± 4.0 | 13 |
|  | *Pre + Post-Gal4 >>*  *TRiP.JF02941*  *GluRIII RNAi* | 0.80 ± 0.06 | 0.3 ± 0.1 | 39.9 ± 1.8 | -69.5 ± 1.1 | 50.6 ± 1.9 *** | 102.0 ± 4.3 *** | 11 |
| *CaMKII* (presynaptic) | *Pre-Gal4* >>  *UAS-CaMKII-Ala* | 0.90 ± 0.03 | 2.0 ± 0.2 | 38.3 ± 1.5 | -69.3 ± 1.0 | 43.2 ± 1.9 | 85.0 ± 5.4 | 15 |
|  | *Pre-Gal4* >>  *UAS-CaMKII-Ala, GluR/GluR* | 0.51 ± 0.02 | 0.8 ± 0.1 | 28.3 ± 1.7 | -67.4 ± 1.1 | 56.6 ± 3.5 ** | 91.1 ± 7.2 | 11 |
|  | *Pre-Gal4* >>  *UAS-CaMKII-Ala*  *+ PhTox* | 0.53 ± 0.02 | 1.2 ± 0.1 | 31.9 ± 2.6 | -66.5 ± 1.0 | 62.1 ± 6.3 *** | 113.66 ± 16.33 | 9 |

| **FIGURE 2 – *IP_3_-sponge* Screen Data and PhTox Challenge** | | | | | | | | |
| --- | --- | --- | --- | --- | --- | --- | --- | --- |
| **Condition** | **Genotype or Reagent** | **mEPSP (mV)** | **mEPSP freq. (Hz)** | **EPSP (mV)** | **V_m_ (mV)** | **QC** | **NLSC QC** | **n** |
| *UAS-IP_3_-sponge.m49* | *Pre + Post-Gal4 >>*  *UAS-IP3-sponge.m49* | 0.89 ± 0.04 | 1.3 ± .01 | 43.1 ± 1.7 | -69.3 ± 1.3 | 49.1 ± 2.0 | 111.1 ± 8.7 | 13 |
|  | *Pre + Post-Gal4 >>*  *UAS-IP_3_-sponge.m49 + GluRIII* RNAi | 0.55 ± 0.03 | 0.3 ± 0.1 | 28.5 ± 1.4 | -64.4 ± 0.5 | 52.4 ± 2.4 | 86.4 ± 5.2 * | 14 |
| *UAS-IP_3_-sponge.m30* | *Pre + Post-Gal4 >>*  *UAS-IP_3_-sponge.m30* | 0.81 ± 0.04 | 1.4 ± 0.2 | 43.8 ± 1.4 | -69.4 ± 1.6 | 55.1 ± 1.8 | 123.3 ± 4.6 | 16 |
|  | *Pre + Post-Gal4 >>*  *UAS-IP_3_-sponge.m30 + GluRIII* RNAi | 0.63 ± 0.02 | 0.3 ± 0.0 | 34.6 ± 1.1 | -67.2 ± 0.9 | 55.5 ± 2.3 | 102.0 ± 5.4 ** (down) | 15 |
| *UAS-IP_3_-sponge.m49*  (PhTox) | *Pre + Post-Gal4 >>*  *UAS-IP_3_-sponge.m49*  (PhTox Cont) | 0.90 ± 0.03 | 1.6 ± 0.2 | 40.1 ± 1.3 | -67.4 ± 1.0 | 45.2 ± 2.4 | 96.5 ± 7.8 | 13 |
|  | *Pre + Post-Gal4 >>*  *UAS-IP_3_-sponge.m49*  PhTox | 0.54 ± 0.02 | 1.0 ± 0.1 | 38.6 ± 2.4 | -65.3 ± 0.9 | 73.1 ± 5.0 *** | 163.2 ± 17.6 ** | 15 |
| *UAS-IP_3_-sponge.m49* | *Pre + Post-Gal4 >>*  *UAS-IP_3_-sponge.m49 + GluRIII* RNAi | 0.66 ± 0.03 | 0.4 ± 0.1 | 31.7 ± 1.2 | -64.9 ± 0.7 | 48.9 ± 1.8 | 86.4 ± 4.7 | 16 |
|  | *Pre + Post-Gal4 >>*  *UAS-IP_3_-sponge.m49 + GluRIII* RNAi  PhTox | 0.40 ± 0.02 | 0.2 ± 0.0 | 27.5 ± 1.8 | -64 ± 0.7 | 69.9 ± 4.9 *** | 116.1 ± 12.1 * | 14 |
