# Supplementary material for: Maintenance of homeostatic plasticity at the Drosophila neuromuscular synapse requires continuous IP3-directed signaling
Source: eLife. 2019 Jun 10;8:e39643. doi: 10.7554/eLife.39643 (PMC6557630; doi:10.7554/eLife.39643)
Supplement: Supplementary file 3. — Genotypes and/or conditions are denoted. Average values ± SEM are presented for each electrophysiological parameter, with n = number of NMJs recorded. Values include miniature excitatory postsynaptic potential (mEPSP) amplitude, mEPSP frequency (Freq), excitatory postsynaptic potential (EPSP) amplitude, quantal content (QC), and QC corrected for non-linear summation (NLS). *p<0.05, **p<0.01, ***p<0.001 vs. unchallenged control. [file elife-39643-supp3.docx]

**Supplementary File 3**

| **FIGURE 4** | | | | | | | | |
| --- | --- | --- | --- | --- | --- | --- | --- | --- |
| **Condition** | **Genotype or Reagent** | **mEPSP (mV)** | **mEPSP freq. (Hz)** | **EPSP (mV)** | **V_m_ (mV)** | **QC** | **NLSC QC** | **n** |
| wild type | 5 μM Xestospongin C | 0.85 ± 0.04 | 3.1 ± 0.3 | 38.8 ± 1.1 | -66.4 ± 0.9 | 46.3 ± 2.3 | 95.2 ±0.5 | 13 |
| *GluRIIA^SP16^* | 5 μM Xestospongin C | 0.46 ± 0.03 | 0.8 ± 0.1 | 28.5 ± 1.9 | -68.2 ± 1.5 | 64.0 ± 4.3 * | 104.3 ± 9.8 | 15 |
| wild type | 20 μM Xestospongin C | 1.00 ± 0.04 | 6.1 ± 0.4 | 37.3 ± 0.8 | -64.1 ± 0.6 | 37.7 ± 0.9 | 76.4 ± 2.5 | 14 |
| wild type | 20 μM PhTox + 20 μM Xestospongin C | 0.56 ± 0.01 | 1.5 ± 0.2 | 34.2 ± 1.0 | -64.6 ± 0.8 | 61.8 ± 1.6 *** | 115.9 ± 4.9 *** | 19 |
| *GluRIIA^SP16^* | 20 μM Xestospongin C | 0.54 ± 0.02 | 1.3 ± 0.2 | 21.4 ± 0.9 | -64.1 ± 0.1 | 40.4 ± 2.1 | 57.4 ± 3.5 *** (down) | 13 |
| wild type | DMSO (2% v/v) | 0.85 ± 0.04 | 5.4 ± 0.5 | 35.8 ± 1.2 | -64.9 ± 0.8 | 42.8 ± 1.8 | 83.1 ± 4.5 | 12 |
| *GluRIIA^SP16^* | DMSO (2% v/v) | 0.46 ± 0.02 | 1.21 ± 0.16 | 26.1 ± 1.1 | -65.2 ± 0.7 | 57.5 ± 3.4 *** | 89.5 ± 7.0 | 11 |
| wild type | PhTox + DMSO (2% v/v) | 0.56 ± 0.02 | 1.9 ± 0.2 | 35.7 ± 1.0 | -67.0 ± 1.1 | 64.5 ± 2.6 *** | 121.2 ± 5.7 *** | 15 |
