# Supplementary material for: Maintenance of homeostatic plasticity at the Drosophila neuromuscular synapse requires continuous IP3-directed signaling
Source: eLife. 2019 Jun 10;8:e39643. doi: 10.7554/eLife.39643 (PMC6557630; doi:10.7554/eLife.39643)
Supplement: Supplementary file 4. — Genotypes and/or conditions are denoted. Average values ± SEM are presented for each electrophysiological parameter, with n = number of NMJs recorded. Values include miniature excitatory postsynaptic potential (mEPSP) amplitude, mEPSP frequency (Freq), excitatory postsynaptic potential (EPSP) amplitude, quantal content (QC), and QC corrected for non-linear summation (NLS). *p<0.05, **p<0.01, ***p<0.001 vs. unchallenged control. [file elife-39643-supp4.docx]

**Supplementary File 4**

| **FIGURE 5** | | | | | | | | |
| --- | --- | --- | --- | --- | --- | --- | --- | --- |
| **Condition** | **Genotype or Reagent** | **mEPSP (mV)** | **mEPSP freq. (Hz)** | **EPSP (mV)** | **V_m_ (mV)** | **QC** | **NLSC QC** | **n** |
| wild type  (GluR cont) | 1 μM 2-APB | 0.80 ± 0.02 | 3.6 ± 0.3 | 35.9 ± 1.3 | -68.0 ± 1.1 | 45.0 ± 1.3 | 85.2 ± 4.3 | 15 |
| *GluRIIA^SP16^* | 1 μM 2-APB | 0.53 ± 0.02 | 0.9 ± 0.1 | 23.4 ± 1.1 | -67.2 ± 0.8 | 45.3 ± 2.6 | 66.4 ± 5.1 ** (down) | 14 |
| wild type (PhTox cont) | 1 μM 2-APB | 0.75 ± 0.02 | 2.9 ± 0.2 | 39.9 ± 1.2 | -66.0 ± 0.9 | 53.5 ± 1.8 | 114.2 ± 6.0 | 11 |
| wild type | 1 μM 2-APB  20 μM PhTox | 0.48 ± 0.04 | 1.6 ± 0.3 | 35.6 ± 1.8 | -63.2 ± 0.5 | 76.9 ± 4.0 *** | 152.3 ± 9.5 ** | 12 |
| wild type | 10 μM 2-APB | 0.83 ± 0.03 | 4.7 ± 0.9 | 43.3 ± 0.8 | -65.0 ± 0.7 | 53.3 ± 2.5 | 128.3 ± 8.1 | 15 |
| *GluRIIA^SP16^* | 10 μM 2-APB | 0.44 ± 0.02 | 0.7 ± 0.1 | 23.9 ± 1.0 | -64.9 ± 0.6 | 54.9 ± 2.9 | 82.2 ± 5.8 *** (down) | 14 |
