# Supplementary material for: Maintenance of homeostatic plasticity at the Drosophila neuromuscular synapse requires continuous IP3-directed signaling
Source: eLife. 2019 Jun 10;8:e39643. doi: 10.7554/eLife.39643 (PMC6557630; doi:10.7554/eLife.39643)
Supplement: Supplementary file 5. — Genotypes and/or conditions are denoted. Average values ± SEM are presented for each electrophysiological parameter, with n = number of NMJs recorded. Values include miniature excitatory postsynaptic potential (mEPSP) amplitude, mEPSP frequency (Freq), excitatory postsynaptic potential (EPSP) amplitude, quantal content (QC), and QC corrected for non-linear summation (NLS). *p<0.05, **p<0.01, ***p<0.001 vs. unchallenged control. [file elife-39643-supp5.docx]

**Supplementary File 5**

| **FIGURE 6** | | | | | | | | |
| --- | --- | --- | --- | --- | --- | --- | --- | --- |
| **Condition** | **Genotype or Reagent** | **mEPSP (mV)** | **mEPSP freq. (Hz)** | **EPSP (mV)** | **V_m_ (mV)** | **QC** | **NLSC QC** | **n** |
| wild type | +100 μM Ryanodine | 0.78 ± 0.02 | 3.7 ± 0.3 | 34.6 ± 1.1 | -66.4 ± 0.9 | 45.0 ± 2.2 | 84.1 ± 5.4 | 17 |
| *GluRIIA^SP16^* | +100 μM Ryanodine | 0.50 ± 0.01 | 1.3 ± 0.1 | 25.4 ± 1.1 | -66.2 ± 0.8 | 51.5 ± 2.4 | 78.4 ± 4.8 | 15 |
| wild type | +10 μM Dantrolene | 0.81 ± 0.04 | 3.2 ± 0.2 | 29.2 ± 1.6 | -64.3 ± 0.5 | 37.3 ± 2.4 | 65.0 ± 5.1 | 25 |
| *GluRIIA^SP16^* | +10 μM Dantrolene | 0.46 ± 0.01 | 0.9 ± 0.1 | 15.9 ± 1.3 | -64.8 ± 0.6 | 34.7 ± 2.9 | 46.5 ± 4.7 ** (down) | 27 |
| wild type | +10 μM Dantrolene | 0.91 ± 0.04 | 2.6 ± 0.2 | 34.8 ± 1.6 | -64.2 ± 0.7 | 39.4 ± 2.6 | 76.5 ± 6.5 | 13 |
| wild type | +PhTox  +10 μM Dantrolene | 0.53 ± 0.02 | 1.4 ± 0.2 | 34.4 ± 1.3 | -64.7 ± 0.9 | 66.1 ± 4.8 *** | 126.3 ± 13.7 ** | 10 |
| *GluRIII* RNAi | *Pre + Post-Gal4 >>*  *GluRIII* RNAi/+  10 μM Dantrolene | 0.06 ± 0.02 | 0.5 ± 0.1 | 37.1 ± 1.9 | -66.7 ± 1.7 | 66.9 ± 4.2 | 131.3 ± 11.8 | 8 |
| *GluRIII* RNAi | *Pre + Post-Gal4 >>*  GluRIII RNAi  +20 μM PhTox  +10 μM Dantrolene | 0.39 ± 0.03 | 0.4 ± 0.1 | 36.2 ± 1.5 | -67.4 ± 1.2 | 96.5 ± 6.0 ** | 183.8 ± 13.7 * | 11 |
