# Supplementary material for: Maintenance of homeostatic plasticity at the Drosophila neuromuscular synapse requires continuous IP3-directed signaling
Source: eLife. 2019 Jun 10;8:e39643. doi: 10.7554/eLife.39643 (PMC6557630; doi:10.7554/eLife.39643)
Supplement: Supplementary file 6. — Genotypes and/or conditions are denoted. Average values ± SEM are presented for each electrophysiological parameter, with n = number of NMJs recorded. Values include miniature excitatory postsynaptic potential (mEPSP) amplitude, mEPSP frequency (Freq), excitatory postsynaptic potential (EPSP) amplitude, quantal content (QC), and QC corrected for non-linear summation (NLS). *p<0.05, **p<0.01, ***p<0.001 vs. unchallenged control. [file elife-39643-supp6.docx]

**Supplementary File 6**

| **FIGURE 7** | | | | | | | | |
| --- | --- | --- | --- | --- | --- | --- | --- | --- |
| **Condition** | **Genotype or Reagent** | **mEPSP (mV)** | **mEPSP freq. (Hz)** | **EPSP (mV)** | **V_m_ (mV)** | **QC** | **NLSC QC** | **n** |
| Driver Control | *Pre + Post-Gal4 >>*  +10 μM Dantrolene | 0.73 ± 0.03 | 0.9 ± 0.1 | 39.9 ± 1.8 | -67.6 ± 0.9 | 55.5 ± 2.7 | 118.2 ± 9.5 | 14 |
| *GluRIII* RNAi | *Pre + Post-Gal4 >>*  *GluRIII* RNAi/+  10 μM Dantrolene | 0.61 ± 0.02 | 0.2 ± 0.0 | 31.5 ± 1.0 | -65.5 ± 0.9 | 52.6 ± 2.2 | 90.8 ± 4.7 | 14 |
| Driver Control  *IP_3_ sponge* | *Pre + Post-Gal4 >>*  *UAS-IP_3_-sponge.m49*  10 μM Dantrolene | 0.93 ± 0.03 | 1.6 ± 0.1 | 41.2 ± 1.7 | -67.6 ± 1.4 | 44.5 ± 1.5 | 96.1 ± 5.8 | 12 |
| *GluRIII* RNAi  *IP_3_ sponge* | *Pre + Post-Gal4 >>*  *GluRIII* RNAi/*UAS-IP_3_-sponge.m49*  10 μM Dantrolene | 0.68 ± 0.05 | 0.4 ± 0.1 | 33.3 ± 1.8 | -66.0 ± 1.1 | 50.3 ± 2.1 | 90.5 ± 4.3 | 13 |
| wild type |  | 0.78 ± 0.04 | 1.8 ± 0.2 | 33.7 ± 0.9 | -64.7 ± 0.5 | 44.6 ± 1.8 | 82.4 ± 4.0 | 20 |
| wild type | + PhTox | 0.65 ± 0.03 | 1.4 ± 0.2 | 36.5 ± 1.0 | -65.0 ± 1.1 | 57.2 ± 3.5 ** | 112.0 ± 7.8 ** | 12 |
| Driver control | *Pre + Post-Gal4* | 0.68 ± 0.04 | 2.0 ± 0.8 | 40.1 ± 1.6 | -66.4 ± 1.0 | 61.1 ± 4.0 | 132.9 ± 12.5 | 13 |
| Driver control | *Pre + Post-Gal4*  PhTox  CNS intact | 0.57 ± 0.03 | 0.7 ± 0.8 | 39.8 ± 0.8 | -66.9 ± 1.3 | 72.5 ± 4.4 | 152.7 ± 10.3 | 12 |
| Driver control | *Pre + Post-Gal4*  PhTox  CNS excised | 0.52 ± 0.02 | 0.7 ± 0.1 | 37.8 ± 1.4 | -66.0 ± 1.3 | 72.9 ± 3.6 * | 149.7 ± 12.6 | 12 |
| *UAS-IP_3_-sponge.m49* | *Pre + Post-Gal4 >>*  *UAS-IP_3_-sponge.m49* | 0.83 ± 0.04 | 1.3 ± 0.1 | 40.4 ± 1.2 | -64.9 ± 0.8 | 50.1 ± 202 | 111.2 ± 6.8 | 15 |
| *UAS-IP_3_-sponge.m49* | *Pre + Post-Gal4 >>*  *UAS-IP_3_-sponge.m49*  PhTox  CNS intact | 0.56 ± 0.02 | 1.0 ± 0.1 | 40.4 ± 1.8 | -65.6 ± 1.0 | 72.8 ± 3.8 *** | 161.9 ± 12.8 ** | 13 |
| *UAS-IP_3_-sponge.m49* | *Pre + Post-Gal4 >>*  *UAS-IP_3_-sponge.m49*  PhTox  CNS excised | 0.56 ± 0.04 | 0.7 ± 0.1 | 34.4 ± 1.6 | -64.9 ± 0.9 | 65.0 ± 5.5 * | 125.6 ± 14.7 | 13 |
