# Supplementary material for: Maintenance of homeostatic plasticity at the Drosophila neuromuscular synapse requires continuous IP3-directed signaling
Source: eLife. 2019 Jun 10;8:e39643. doi: 10.7554/eLife.39643 (PMC6557630; doi:10.7554/eLife.39643)
Supplement: Supplementary file 7. — Genotypes and/or conditions are denoted. Average values ± SEM are presented for each electrophysiological parameter, with n = number of NMJs recorded. Values include miniature excitatory postsynaptic potential (mEPSP) amplitude, mEPSP frequency (Freq), excitatory postsynaptic potential (EPSP) amplitude, quantal content (QC), and QC corrected for non-linear summation (NLS). *p<0.05, **p<0.01, ***p<0.001 vs. unchallenged control. [file elife-39643-supp7.docx]

**Supplementary File 7**

| **FIGURE 8** | | | | | | | | |
| --- | --- | --- | --- | --- | --- | --- | --- | --- |
| **Condition** | **Genotype or Reagent** | **mEPSP (mV)** | **mEPSP freq. (Hz)** | **EPSP (mV)** | **V_m_ (mV)** | **QC** | **NLSC QC** | **n** |
| Driver Control  (Neuron) | *Pre-Gal4* | 0.95 ± 0.04 | 2.6 ± 0.2 | 38.7 ± 1.1 | -66.5 ± 1.0 | 41.9 ± 2.3 | 86.2 ± 5.8 | 17 |
|  | *Pre-Gal4*  *GluRIIA^SP16^* | 0.49 ± 0.01 | 1.3 ± 0.2 | 29.5 ± 1.2 | -65.1 ± 0.7 | 60.3 ± 2.2 *** | 101.2 ± 6.1 | 15 |
| *IP_3_-sponge* | *Pre-Gal4 >>*  *UAS-IP_3_-sponge.m49* | 1.09 ± 0.04 | 3.5 ± 0.0 | 40.8 ± 1.5 | -69.9 ± 1.3 | 37.6 ± 1.3 | 77.9 ± 3.7 | 16 |
| *GluRIIA*  *IP_3_-sponge* | *Pre-Gal4 >>*  *UAS-IP_3_-sponge.m49*  *GluRIIA^SP16^* | 0.47 ± 0.01 | 0.6 ± 0.1 | 24.3 ± 2.1 | -68.4 ± 1.1 | 51.6 ± 4.0 ** | 79.2 ± 9.4 | 15 |
| Driver Control  (Muscle) | Post-Gal4 | 0.76 ± 0.0 | 2.8 ± 0.2 | 38.7 ± 1.0 | -67.6 ± 1.0 | 51.6 ± 2.1 | 103.6 ± 5.3 | 14 |
|  | Post-Gal4  *GluRIIA^SP16^* | 0.41 ± 0.01 | 0.7 ± 0.1 | 26.8 ± 1.0 | -66.2 ± 0.8 | 66.7 ± 3.0 *** | 105.4 ± 6.6 | 14 |
| *IP_3_-sponge* | *Post-Gal4* >>  *UAS-IP_3_-sponge.m49* | 0.85 ± 0.03 | 2.1 ± 0.2 | 38.3 ± 1.1 | -65.7 ± 0.7 | 45.4 ± 1.5 | 93.6 ± 4.8 | 15 |
| *GluRIIA*  *IP_3_-sponge* | *Post-Gal4* >>  *UAS-IP_3_-sponge.m49*  *GluRIIA^SP16^* | 0.51 ± 0.03 | 1.4 ± 0.2 | 27.8 ± 1.3 | -65.3 ± 0.9 | 56.2 ± 0.7 ** | 92.1 ± 7.9 | 16 |
