# Supplementary material for: Maintenance of homeostatic plasticity at the Drosophila neuromuscular synapse requires continuous IP3-directed signaling
Source: eLife. 2019 Jun 10;8:e39643. doi: 10.7554/eLife.39643 (PMC6557630; doi:10.7554/eLife.39643)
Supplement: Transparent reporting form [file elife-39643-transrepform.pdf]

eLife's transparent reporting form

We encourage authors to provide detailed information *within their submission* to facilitate the interpretation and replication of experiments. Authors can upload supporting documentation to indicate the use of appropriate reporting guidelines for health-related research (see EQUATOR Network), life science research (see the BioSharing Information Resource), or the ARRIVE guidelines for reporting work involving animal research. Where applicable, authors should refer to any relevant reporting standards documents in this form.

If you have any questions, please consult our Journal Policies and/or contact us: editorial@elifesciences.org.

Sample-size estimation

- You should state whether an appropriate sample size was computed when the study was being designed
- You should state the statistical method of sample size computation and any required assumptions
- If no explicit power analysis was used, you should describe how you decided what sample (replicate) size (number) to use

Please outline where this information can be found within the submission (e.g., sections or figure legends), or explain why this information doesn't apply to your submission:

Statistical methods are found in a section titled "Statistical Analyses" in the Materials and Methods section.

Exact sample sizes for all electrophysiological experiments reported in the text and figures can be found in the Supplementary Files. We did not calculate an exact sample size *a priori*. Instead, based on prior work and power analyses conducted by our lab and other labs studying homeostatic plasticity at the NMJ, we estimated that sample sizes of n = ~ 10-16 NMJs would likely yield sufficient data to test for significant homeostatic compensation (or not) using appropriate two-tailed tests. The exception was our genetic screen described in Figure 2. For the genetic screen for factors that impair the maintenance of homeostatic compensation, some $n < 10$. We recognized that this could result in false negatives for the screen. However, for cases where $n < 10$ for the screen, significant compensation was observed.

After collecting and analyzing data, we ran statistical analyses as detailed in each figure legend. For experiments where statistical significance was achieved ($p < 0.05$), the appropriate range of p values were reported in the figures and legends. For experiments where statistical significance was not achieved, exact p values were reported on the figures.

Replicates

- You should report how often each experiment was performed
- You should include a definition of biological versus technical replication
- The data obtained should be provided and sufficient information should be provided to indicate the number of independent biological and/or technical replicates

- If you encountered any outliers, you should describe how these were handled
- Criteria for exclusion/inclusion of data should be clearly stated
- High-throughput sequence data should be uploaded before submission, with a private link for reviewers provided (these are available from both GEO and ArrayExpress)

Please outline where this information can be found within the submission (e.g., sections or figure legends), or explain why this information doesn't apply to your submission:

All replicates within our data sets are biological replicates (identical genotypes or treatments, but analyses at unique NMJs comprising a data set). Summary electrophysiological data, including numbers of biological replicates, are reported in the Supplementary Files 1-7. Raw data for all figure calculations are reported in the Source Data files linked to each figure (Microsoft Excel .xlsx).

For each new figure, there are new control and experimental data sets to answer new key questions; statistical analyses were run on those data sets. Some baseline control data sets are plotted and re-plotted on multiple figures, strictly for visual comparison. The figure legends indicate when this is the case.

Statistical reporting

- Statistical analysis methods should be described and justified
- Raw data should be presented in figures whenever informative to do so (typically when N per group is less than 10)
- For each experiment, you should identify the statistical tests used, exact values of N, definitions of center, methods of multiple test correction, and dispersion and precision measures (e.g., mean, median, SD, SEM, confidence intervals; and, for the major substantive results, a measure of effect size (e.g., Pearson's r, Cohen's d)
- Report exact p-values wherever possible alongside the summary statistics and 95% confidence intervals. These should be reported for all key questions and not only when the p-value is less than 0.05.

Please outline where this information can be found within the submission (e.g., sections or figure legends), or explain why this information doesn't apply to your submission:

Statistical methods applied are found in a section titled "Statistical Analyses" in the Materials and Methods section and repeated in the Figure legends. Exact *n* values are reported in the Supplementary Files 1-7 and in the figures. Summary statistics and *p* values are reported in the figures and legends. Exact *p* values in which $p > 0.05$ for key questions are also reported in the figures. Raw data for the measures underlying the figures and statistics are reported in the Source Data Excel documents linked to each figure.

(For large datasets, or papers with a very large number of statistical tests, you may upload a single table file with tests, Ns, etc., with reference to sections in the manuscript.)

Group allocation

- Indicate how samples were allocated into experimental groups (in the case of clinical studies, please specify allocation to treatment method); if randomization was used, please also state if restricted randomization was applied
- Indicate if masking was used during group allocation, data collection and/or data analysis

Please outline where this information can be found within the submission (e.g., sections or figure legends), or explain why this information doesn't apply to your submission:

Control and experimental samples were collected in parallel, using identical conditions (i.e. the same batch of recording saline) and were analyzed post-hoc blind to genotype. These descriptions are found in the Materials and Methods section.

Additional data files ("source data")

- We encourage you to upload relevant additional data files, such as numerical data that are represented as a graph in a figure, or as a summary table
- Where provided, these should be in the most useful format, and they can be uploaded as "Source data" files linked to a main figure or table
- Include model definition files including the full list of parameters used
- Include code used for data analysis (e.g., R, MatLab)
- Avoid stating that data files are "available upon request"

Please indicate the figures or tables for which source data files have been provided:

Summary electrophysiological data and relevant parameters for all electrophysiological experiments are shared in the Supplementary Files. Raw data are included in the Source Data Excel files.
